# SKN-1 ISOFORM-C IS ESSENTIAL FOR C. ELEGANS DEVELOPMENT

**DOI:** 10.1093/geroni/igae098.3718

**Published:** 2024-12-31

**Authors:** Marisa Gaglio, Vandita Gorla, Tripti Nair, Carmen Ramos, Chris Turner, Sean Curran

**Affiliations:** University of Southern California, Los angeles, California, United States; University of Southern California, Los Angeles, California, United States; University of Southern California, Los Angeles, California, United States; University of Southern California, Los Angeles, California, United States; University of Southern California, Los Angeles, California, United States; University of Southern California, Los Angeles, California, United States

## Abstract

The transcription factor SKN-1 in Caenorhabditis elegans is a critical regulator of various biological processes, impacting development, diet and immune responses, cellular detoxification, and lipid metabolism; thereby playing a pivotal role in regulating the health and lifespan of the organism. The primary isoforms of SKN-1 (SKN-1a, SKN-1b, and SKN-1c) exhibit distinct functions resembling mammalian Nrf transcription factors. This study investigates the specific role of the SKN-1c isoform in development by generating mutants with targeted missense mutations in the skn-1c and skn-1a isoforms. The skn-1c Met1Ala mutants, which replaces a start methionine with alanine, renders SKN-1c non-functional while preserving other isoforms, produced inviable embryos, requiring a balancer chromosome for proper embryonic development. In contrast, skn-1a Met1Ala mutants, which replaces the start methionine with alanine for this isoform, displayed normal embryonic development and hatching. Moreover, the data suggest that SKN-1c plays a crucial role in embryonic development, as strains without maternally deposited SKN-1c lead to embryos that are developmentally arrested. Together, these findings contribute to our understanding of SKN-1c’s specific role in influencing embryogenesis and development in C. elegans.

